# Assessment of oro-dental manifestations in a series of acromegalic patients, the AcroDent study

**DOI:** 10.1530/EC-20-0176

**Published:** 2020-07-30

**Authors:** Sylvain Roumeau, Joannice Thevenon, Lemlih Ouchchane, Salwan Maqdasy, Marie Batisse-Lignier, Christian Duale, Nathalie Pham Dang, Philippe Caron, Igor Tauveron, Laurent Devoize

**Affiliations:** 1 CHU de Clermont-Ferrand, Service d’Endocrinologie, Diabétologie et Maladies Métaboliques, Clermont-Ferrand, France; 2 Université Clermont Auvergne, Faculté de Médecine, Clermont-Ferrand, France; 3 CHU Clermont-Ferrand, Service d’Odontologie, Clermont-Ferrand, France; 4 Université Clermont Auvergne, CNRS, ISIT, Clermont-Ferrand, France; 5 CHU Clermont-Ferrand, Service de Biostatistiques, Clermont-Ferrand, France; 6 CHU de Clermont-Ferrand, Service d’Endocrinologie, Diabétologie et Maladies Métaboliques, Clermont-Ferrand, France; 7 Laboratoire GReD: UMR Université Clermont Auvergne-CNRS 6293, INSERM U1103, Clermont-Ferrand, France; 8 Université Clermont Auvergne, Inserm, Neuro-Dol, Clermont-Ferrand, France; 9 CHU Clermont-Ferrand, Inserm CIC 1405, Clermont-Ferrand, France; 10 CHU de Clermont-Ferrand, Service de chirurgie maxillo-faciale, Clermont-Ferrand, France; 11 CHU Larrey-Rangueil, Service Endocrinologie et Maladies Métaboliques, Pôle Cardio-Vasculaire et Métabolique, Toulouse, France; 12 Université Clermont Auvergne, Faculté de Chirurgie Dentaire, Clermont-Ferrand, France

**Keywords:** acromegaly, growth hormone-secreting pituitary adenoma, oral manifestations, periodontal diseases, quality of life

## Abstract

**Objective:**

The dental and periodontal impact of GH/IGF-1 hypersecretion has been poorly investigated until now. Our aim is to precisely describe the oro-dental state of acromegalic patients and to study the impact of GH/IGF-1 hypersecretion on patients’ reported oral health-related quality of life (OHRQoL).

**Methods:**

After collecting characteristics of their disease, acromegalic patients answered the GOHAI questionnaire assessing their OHRQoL, the AcroQoL questionnaire and then benefited from a complete stomatological and radiological examination (orthopantomogram systematically, retro-alveolar radiography or Cone Beam CT if necessary).

**Results:**

In total, 29 patients aged 59.1 ± 16.0 years were included. The average DMFT index (sum of Decayed, Missing and Filled Teeth per patient) was 19.0 ± 7.8. 16/29 patients had a gingivitis and 18/29 a mild to moderate chronic periodontitis, but no case of severe chronic periodontitis was found, probably because the frequency of a protective thick gingival biotype was increased (9/29). No case of generalized gingival hypertrophy or diffuse hypercementosis was observed. According to the Add-GOHAI score, only 8/26 patients had a satisfactory OHRQoL. This parameter was correlated to the acromegaly-specific quality of life according to the AcroQoL score. Interestingly, 11/29 patients had bulky oral bony outgrowths (OBO), such as large maxillary or mandibular tori and multiple vestibular exostosis.

**Conclusions:**

The unsatisfactory OHRQoL reported by acromegalic patients contrasts with a rather good objective oro-dental state and annual oral examination seems relevant in this population. Finally, we report that huge OBO could be helpful signposts for the diagnosis of acromegaly.

## Introduction

Oral and maxillofacial manifestations of acromegaly are hallmarks of the disease, including prognathism, inter-dental space enlargement (diastema), occlusion disorder (class III), temporo-mandibular joint pain, macroglossia and obstructive sleep apnea (1, 2, 3, 4, 5, 6). They can develop precociously as oral symptoms were reported to be the first symptoms of the disease by 10% of the patients and to be present at diagnosis for more than 20% of them (7). However, many aspects of the oral condition in this pathology have not been properly described. Indeed, the consequences of elevated GH and IGF-1 on gingivae is debated. Some observations describe a gingival enlargement in acromegalic patients (8), which is known to promote periodontal disease leading to tooth loss, while others suggest that acromegaly could protect against this frequent medical condition (9, 10, 11). Besides, two cases reports described hypercementosis (overgrowth of the cement) in an acromegalic patient (12, 13) but the link with the disease remains questionable. Most importantly, the dental state and the impact of oral manifestations on the patient’s quality of life are not reported at all in acromegaly. Therefore, we conducted a prospective study which aims to establish the precise oral health status in acromegalic patients treated in our tertiary center, with particular regards to the dental and periodontal condition, and oral health related quality of life (OHRQoL).

## Methods

### Study population

A total of 29 adult patients with acromegaly undergoing treatment or long-term follow-up in the endocrinology department of the University Hospital of Clermont-Ferrand, France, were prospectively enrolled to assess their oral health and OHRQoL between December 2016 and April 2018. Patients had to be previously or newly diagnosed with acromegaly according to the French recommendations (14) and to have an available recent evaluation of the clinical, biological and morphological characteristics of the disease. Patients with a medical history of pathologies or treatments leading to significant oral alterations (such as treatments known to induce gingival hypertrophy) were excluded.

Demographic characteristics, acromegaly history and last disease assessment results were obtained from patient’s medical records. GH and IGF-1 levels were determined at the time of inclusion in the department of biochemistry of the University Hospital of Clermont-Ferrand, France, with automated chemiluminescence immunoassay (Immulite 2000, Siemens, Erlangen, Germany) and used to classify hormonal control according to current recommendations (14).

All patients answered AcroQoL and EPICES auto-questionnaires. AcroQoL is a validated disease-specific questionnaire to assess health-related quality of life (HRQoL) in patients with acromegaly (15). It is composed of 22 questions answered on a 5-point Likert scale (no symptoms = 5 points, severe symptoms = 1 point). The total score corresponds to the sum of all the points then rescored on a 0–100 basis, (best score = 110 points = 100%, worst score = 22 points = 0%). A total score below 80 points, that is, 66% is considered as a clinically significant impact of acromegaly on patients’ quality of life. This score can be divided into three dimensions: Physical Symptoms subscale (i.e., Items 1, 3, 9, 13–15, 19, and 22), Appearance Issues subscale (i.e., Items 2, 4, 7, 11, 12, 16, and 17), and Personal Relation Issues subscale (Items 5, 6, 8, 10, 18, 20 and 21). As for the global score, results for each dimension were scored on a 0 to 100 basis. Only fully completed AcroQoL questionnaires were included in the analyses and all results (total score and dimensions) are presented as percent. EPICES (Evaluation de la Précarité et des Inégalités de santé dans les Centres d’Examens de Santé) is a French questionnaire assessing socio-economic conditions (16). It is made up of 11 ‘yes/no’ questions, the answers of which make it possible to calculate a score between 0 (lack of insecurity) and 100 (maximum precariousness). A score above 30 is linked to poor healthcare accessibility, especially for dental care in the French population (16).

### Oral examination

All patients underwent an oral examination and an orthopantomogram in the odontology department of the University Hospital of Clermont-Ferrand, France. All examinations and radiological analysis were performed by the same experimented specialist. The DMFT index (sum of the number of Decayed, Missing and Filled Teeth) was determined. Probing was performed at six sites for each tooth to assess gingival sulcus depth, pocket presence and depth, bleeding on probing and plaque presence. Gingival biotype and gingival hypertrophy, as well as all other oral lesions, were noted. As recommended by the American Academy of Periodontology, periodontitis was defined by the presence of gingival pockets, that is, gingival depth sulcus greater than 4 mm, which leads to an inflammatory zone evolving to a loss of dental attach and finally to a tooth loss (17). Presence of pockets deeper than 6 mm defined severe periodontitis. If pockets affected less than 30% of the teeth present in the oral cavity, *localized* chronic adult periodontitis was diagnosed. In this case, it is likely that lesions result from the difficulty of teeth cleaning in a particular zone. On the opposite, the diagnosis of *generalized* chronic adult periodontitis was retained (17). Bleeding on probing on a tooth without associated gingival pocket was classified as gingivitis. Finally, ‘healthy gums’ means total absence of gingival hypertrophy, gingivitis and periodontitis. Hypercementosis and all other radiological abnormalities were described using orthopantomogram, and if necessary retro-alveolar radiography or Cone Beam CT (CBCT).

### Assessment of oral health-related quality of life (OHRQoL)

OHRQoL was assessed by the self-administered GOHAI questionnaire (General Oral Health Assessment Index) (18, 19). It consists of 12 questions, and each is answered on a 5-point Likert scale, exploring dimensions of physical functions (eating, speaking and swallowing), psycho-social impact of oral health and pain or discomfort. Two scores were calculated from the answers: the Add-GOHAI which is the sum of all points and the simple-count GOHAI (SC-GOHAI) which is the number of symptoms referred to as ‘sometimes’, ‘often’ or ‘always’, which are thus clinically significant (19). Satisfactory OHRQoL is achieved by an Add-GOHAI score ≥ 57, moderate OHRQoL by an Add-GOHAI score between 50 and 57 and poor OHRQoL by an Add-GOHAI score ≤ 50 (18). For SC-GOHAI scores rank from 0 (no significant symptom) to 12 (worst).

### Ethical approval

This study was approved by the local Medical Ethical Review Committee (reference number DC-2015-2462), and registered on ClinicalTrials.gov (number NCT03401008). All the participants provided a written informed consent.

### Statistical analysis

Continuous variables are presented as means ± s.d. and categorical variables as frequencies and proportions. Means comparisons between groups were performed using unpaired Student’s *t*-test, with Welch correction when appropriate. Comparisons of categorical variables and proportions were performed using the Fisher exact test. Pearson’s linear correlation coefficients were calculated to test correlations between quantitative variables. All statistical analyses were performed with a double-sided type I error set at 5%, using SAS v9.4 (SAS Institute Inc., Cary, NC, USA).

## Results

### Study population

Twenty-nine acromegalic patients were enrolled. Their characteristics are summarized in Table 1. Mean age was 59.1 ± 16.0 years and 13/29 were males. At inclusion, 14 patients had active uncontrolled acromegaly. On average patients displayed more than three chronic GH excess-related complications (among diabetes, hypertension, dyslipidemia, cardiovascular, joints, gut and thyroid complications) and 28 patients presented at least one. Average AcroQoL score was 69.2 ± 16.5%, meaning globally a moderate alteration of the HRQoL linked to acromegaly, and EPICES score was 13.3 ± 15.4%, suggesting that oral problems detected during oral examination are not due to a socio-economic difficulty of access to care.

**Table 1:** General characteristics of the included patients.

Variables	Value/number
Age (years)	59.1 ± 16.0
Sex (M/F)	13/16
Time from diagnosis (years)	13.3 ± 12.9
Initial MRI: micro/macro-adenoma (*n* = 26)	5/21
IGF-1 at diagnosis (UNL, *n* = 15)	2.89 ± 1.57
Patients status	
Controlled, *n*	15
*Cured, n*	7
*Normalized without treatment, n*	4
*Normalized with current treatment, n*	4
Uncontrolled , *n*	14
Current GH (ng/mL, *n* = 27)	2.07 ± 2.50
Current IGF-1 *all patients* (UNL, *n* = 29)	1.02 ± 0.59
*IGF-1 controlled *(UNL, *n = *15)	0.57 ± 0.26
*IGF-1 uncontrolled *(UNL, *n = *14)	1.50 ± 0.44
Previous treatment	
Pituitary surgery, *n*	22
Pituitary radiotherapy, *n*	5
Medical treatment, *n*	20
*Dopamine agonist, n*	7
*Somatostatin receptor ligands, n*	20
*GH receptor antagonist, n*	4
Never treated, *n*	3
Complications	
Number of complications per patient	3.7 ± 1.7
Diabetes, *n*	9
Hypertension, *n*	13
Dyslipidemia, *n*	14
Cardiovascular complications, *n*	12
Joints complications, *n*	20
Gut complications, *n*	21
Thyroid complications, *n*	18
Pituitary failure, *n* (at least one axis)	12
AcroQoL total score (%, *n* = 24)	69.2 ± 16.5
Physical dimension (%, *n* = 24)	70.3 ± 19.6
Appearance dimension (%, *n* = 24)	58.3 ± 21.3
Personal relation dimension (%, *n* = 24)	78.7 ± 16.4
EPICES score (*n* = 25)	13.3 ± 15.4

Number of assessed patients: *n* = 29 except otherwise mentioned. Data are given as absolute numbers or mean ± s.d. IGF-1 results are expressed as the fold increase of the upper normal limit (UNL) of the test for each patient.

F, female; M, male.

### Dental and periodontal assessment

None of the patients was edentulous. The number of tooth decays (D), missing teeth (M) and filled teeth (F) per patient was 0.48 ± 0.82, 9.4 ± 7.8 and 9.1 ± 4.8, respectively (Table 2). The DMFT index for all patients was 19.0 ± 7.8 and it was positively and strongly linked to age (Pearson’s coefficient = 0.71; *P* < 0.0001).

**Table 2:** Dental and periodondal characteristics of the acromegalic patients.

Variables	Value/number
Dental assessment	
Number of tooth decays per patient (D)	0.48 ± 0.82
Number of missing teeth per patient (M)	9.4 ± 7.8
Number of filled teeth per patient (F)	9.1 ± 4.8
DMFT Index (D + M + F)	19.0 ± 7.8
Periodontal assessment	
Healthy gums, *n*	4
All gingivitis, *n*	16
Isolated gingivitis, *n*	7
Gingivitis associated with localized periodontitis elsewhere, *n*	9
All chronic periodontitis, *n*	18
Localized chronic periodontitis, *n*	13
Generalized chronic periodontitis, *n*	5
Severe chronic periodontitis , *n*	0
Pocket depth (mm)	4.5 ± 0.5
Number of teeth with pockets in patients with periodontitis (*n* = 17)	4.4 ± 3.6
Sites with bleeding on probing^a^ (%)	6.5 ± 7.8
Site with dental plaque^a^ (%)	14 ± 21
Gingival biotype	
Thin, *n*	1
Medium, *n*	19
Thick, *n*	9
Gingival hypertrophy*, n*	1
Hypercementosis*, n*	2
Oral bony outgrowths	
Mandibular tori, *n*	4
Maxillary tori, *n*	4
Vestibular exostosis, *n*	3
All patient with bony outgrowths, *n*	11

Number of assessed patients: *n* = 29. Probing was performed at six sites for each tooth to assess pocket presence and depth, bleeding on probing and plaque presence. Data are given as absolute numbers or mean ± s.d.

^a^Frequency expressed as a percentage of all probed sites per patient.

**Figure 1:**
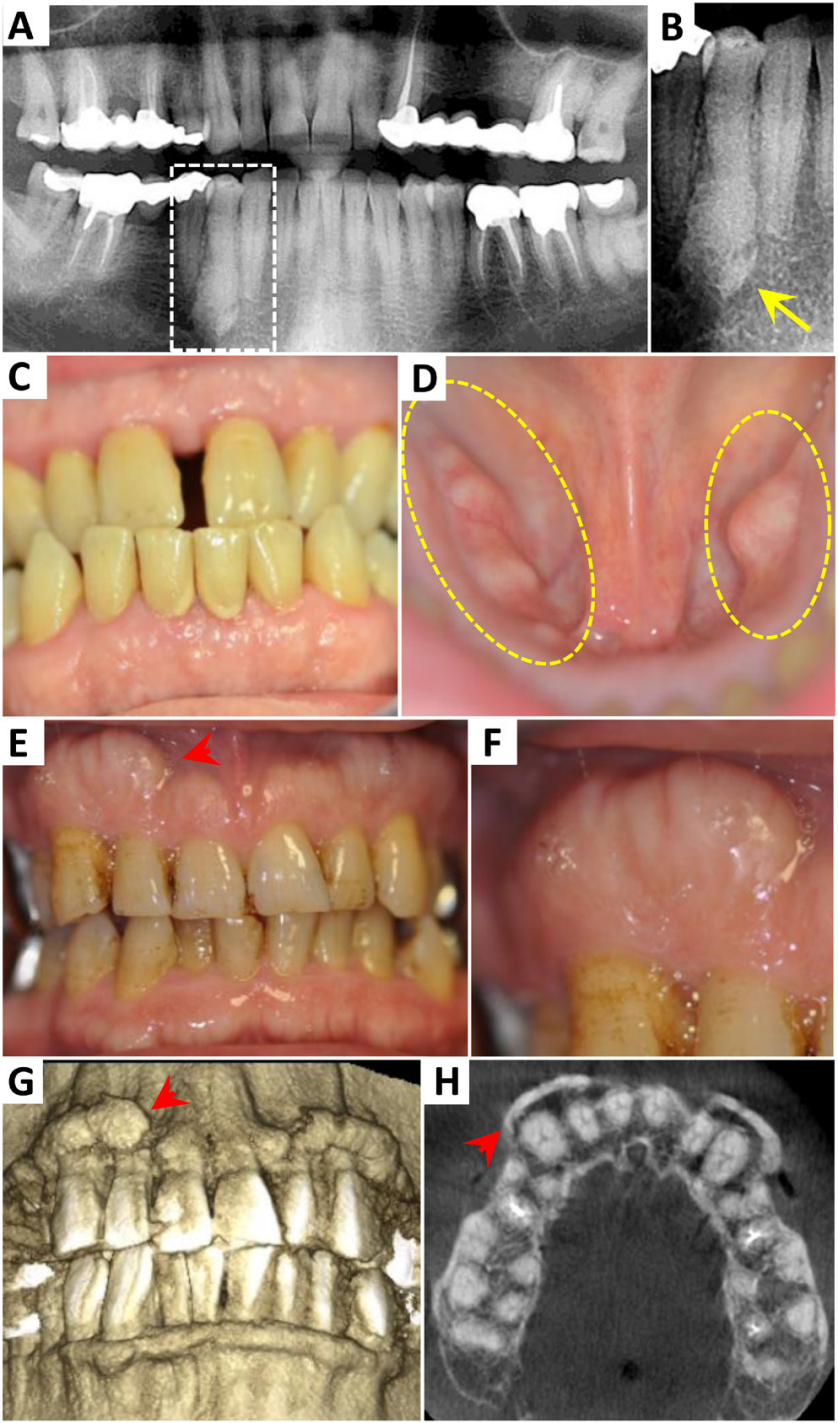
Acromegalic patients had frequent thick gingival biotype and oral bony outgrowths but no diffuse hypercementosis. (A) Orthopantomogram of an acromegalic patient recovering an isolated cementoma of the tooth 44 but no diffuse hypercementosis. (B) Enlargement of the dotted line delimited area in A, yellow arrow: cementoma. (C, D and F) Photographs of oral cavity of acromegalic patients showing: (C) thick gums without gingival hypertrophy nor periodontitis. Note the maxillary central diastema and occlusion disorder (class III); (D) presence of bilateral mandibular tori (circled in dotted line); (E) presence of vestibular bony outgrowths; (F) magnification of the bony outgrowth pointed by a red arrowhead in E; (G) 3D reconstruction from Cone beam CT (CBCT) of the same patient as in E; (H) CBCT slice through maxillary of the same patient as in E. Red arrow heads in E, G and H point to the same vestibular bony outgrowth, note the cortical bone enlargement H.

Concerning gingival state, 4/29 patients had healthy gums, 16/29 had isolated gingivitis, 13/29 had localized chronic adult periodontitis and 5/29 had generalized chronic adult periodontitis (Table 2). No case of severe periodontitis was found. The average number of teeth with pockets in patients with periodontitis was 4.4 ± 3.6 and mean pocket depth was 4.5 ± 0.5 mm, arguing for globally mild disease. Bleeding on probing was present only in 6.5 ± 7.8% of probed sites and dental plaque in 14 ± 24% of probed sites, reflecting a satisfactory oral hygiene. Only one patient had gingival hypertrophy, which was localized as a bilateral palatal tuberous outgrowth. No case of diffuse gingival hypertrophy was detected. Hypercementosis was detected in 2/29 patients and was localized on a unique tooth in each case, as cementomas (Fig. 1A and B). Diffuse enlargement of the cement was never observed on patient’s orthopantomograms. Interestingly, as many as 9/29 patients had a thick gingival biotype while 19/29 had a medium gingival biotype and only 1/29 had a thin gingival biotype (Fig. 1C).

### Presence of oral bony outgrowths (OBO)

Oral examination revealed bulky bony outgrowths in 11/29 acromegalic patients. Large tori which are asymptomatic bony outgrowths on the bony palate (maxillary tori) or lingual face of mandible (mandibular tori, Fig. 1D) and vestibular exostosis (Fig. 1E, G and H) were observed, while these manifestations are not common in the general population. Half of the bony outgrowths measured between 3 and 6 mm in their longer axis and the other half was bigger than 6 mm. Comparison between characteristics of patient subgroups with (*n* = 11/29) or without (*n* = 18/29) OBO is shown in Table 3. Patients with OBO were younger (51.8 ± 15.5 years vs 63.6 ± 15.1 years, *P* = 0.05) and there was no significant difference for sex, time from diagnosis, number of complications, current IGF-1 and proportion of controlled patients between subgroups. Presence of OBO had no impact on the prevalence of thick gingival biotype, gingivitis or periodontitis. The DFMT index was not different in patients with or without OBO, which argues for a similar dental state in both subgroups.

**Table 3:** Characteristics of patients according to the presence of oral bony outgrowths (OBO).

**Variables**	Presence of OBO (*n* = 11)	Absence of OBO (*n* = 18)	*P* value
Age (years)	51.8 ± 15.5	63.6 ± 15.1	0.05
Male,* n*	6	7	0.47
Time from diagnosis (years)	12.6 ± 12.1	13.7 ± 13.7	0.84
Number of complications	3.5 ± 1.8	3.8 ± 1.6	0.72
Disease control, *n*	7	8	0.45
Current GH (ng/mL)	1.52 ± 0.36	2.39 ± 0.90	0.39
Current IGF-1 (UNL)	0.89 ± 0.53	1.10 ± 0.62	0.37
Total AcroQoL (%)	78.0 ± 13.8	63.9 ± 16.0	0.04
EPICES	16.0 ± 16.1	11.2 ± 15.1	0.37
DFMT index	17.9 ± 8.3	19.7 ± 7.6	0.55
Gingivitis,* n*	7	9	0.70
Periodontitis, *n*	5	6	0.61
Thick gingival biotype, *n*	5	4	0.24

Data are given as absolute numbers or mean ± s.d. For statistical tests see ‘Methods’ section.

OBO, oral bony outgrowths; UNL, fold of the Upper Normal Limit.

### Oral health-related quality of life

The GOHAI questionnaire was fully completed by 26/29 patients. The item ‘How often were you worried or concerned about the problems with your teeth, gums or dentures?’ resulted in the least satisfactory answer, and the dimension ‘pain or discomfort’ was more altered than the dimensions ‘physical functions’ and ‘psycho-social functions’ in acromegalic patients. The average Add-GOHAI score was 50.9 ± 8.0 and the average SC-GOHAI score was 3.4 ± 3.1. Based on Add-GOHAI score, 8/26 patients had a satisfactory OHRQoL, 9/26 a moderate OHRQoL and 9/26 a poor OHRQoL. Add-GOHAI and SC-GOHAI scores were strongly correlated with the AcroQoL score (Pearson’s coefficient = 0.52 and −0.54; *P* < 0.01 and *P* < 0.01, respectively), but there were no significant correlations between GOHAI scores and age, the number of acromegalic complications, current IGF-1, DFMT index and EPICES score. Analysis of Add-GOHAI score distribution revealed a clear bimodal repartition as no patient had a score between 45 and 52. We then performed a *post hoc* analysis to compare characteristics of patients with *unaltered* OHRQoL (Add-GOHAI score >52, *n* = 17/26) and patients with *altered* OHRQoL (Add-GOHAI score <45, *n* = 9/26) to seek for factors linked to OHRQoL alteration in acromegaly (Table 4). There was no significant difference between these groups in age, sex, EPICES score, disease duration or control. Oral findings were not different between these groups but acromegaly-specific quality of life assessed by AcroQoL total score was significantly lower in the poor OHRQoL group compared to the satisfactory or moderate OHRQoL group (56.6 ± 17.6% vs 75.1 ± 13.3%, respectively, *P* < 0.01). All three dimensions of AcroQoL were affected, but the difference between groups was more marked for physical symptoms and appearance issues than for personal relation issues (Table 4).

**Table 4:** Characteristics of patients according to the presence of an alteration of the oral health-Related Quality of Life (OHRQoL).

**Variables**	Unaltered OHRQoL (*n* = 17)	Altered OHRQoL (*n* = 9)	*P* value
Age (years)	56.8 ± 14.2	61.2 ± 17.7	0.49
Male,* n*	8	5	0.99
Time from diagnosis (years)	13 ± 13.3	13.9 ± 12.5	0.87
Number of complications	3.9 ± 1.6	3.7 ± 1.6	0.75
Diabetes, *n*	6	3	0.99
Disease control, *n*	9	4	0.99
Current GH (ng/mL)	1.75 ± 1.84	2.71 ± 4.08	0.57
Current IGF-1 (UNL)	1.01 ± 0.55	1.13 ± 0.71	0.63
Total AcroQoL (%)	75.1 ± 13.3	56.6 ± 17.6	0.01
Physical dimension (%)	76.6 ± 15.8	58.0 ± 23.1	0.03
Appearance dimension (%)	66.1 ± 19.1	41.8 ± 18.6	0.01
Personal relation dimension (%)	82.4 ± 14.7	69.9 ± 19.1	0.10
EPICES	10.9 ± 16.9	20 ± 12.3	0.19
DFMT index	19.5 ± 6.9	18.6 ± 8.4	0.75
Gingivitis,* n*	10	6	0.99
Periodontitis, *n*	10	6	0.99
Thick gingival biotype, *n*	7	1	0.19
OBO presence, *n*	9	2	0.22

Unaltered OHRQoL: Add-GOHAI score >52, Altered OHRQoL (Add-GOHAI score <45). Data are given as absolute numbers or mean ± s.d. For statistical tests see ‘Methods’ section. AcroQoL questionnaire was fully completed by 16/17 patients in the Unaltered OHRQoL subgroup and by 7/9 patients in the altered OHRQoL subgroup.

OBO, oral bony outgrowths; OHRQoL, Oral Health-Related Quality of Life; UNL, fold of the upper normal limit.

### Effect of disease control on oral health and related quality of life

Comparison between characteristics of controlled (*n* = 15/29) and uncontrolled patients (*n* = 14/29) is shown in Table 5. There was no significant difference concerning age, sex, time from diagnosis and number of complications between subgroups. The DFMT index, the OHRQoL (GOHAI scores) and the AcroQoL score were not linked to acromegaly control and there was no correlation between IGF-1 level and AcroQoL score (Pearson’s coefficient = −0.16; *P* = 0.45). Control of acromegaly had no effect on the frequency of thick gingival biotype, gingivitis or periodontitis.

**Table 5:** Characteristics of patients according to disease control.

**Variables**	Controlled acromegaly (*n* = 15)	Uncontrolled acromegaly (*n* = 14)	*P* value
Age (years)	60.9 ± 18.6	57.3 ± 13.2	0.56
Male,* n*	6	7	0.72
Time from diagnosis (years)	15.2 ± 12.6	11.3 ± 13.1	0.21
Number of complications	3.3 ± 1.5	4.1 ± 1.8	0.24
Current GH (ng/mL)	1.00 ± 0.99	3.22 ± 3.12	0.03
Current IGF-1 (UNL)	0.57 ± 0.26	1.50 ± 0.44	<0.0001
Total AcroQoL (%)	71.2 ± 14.0	67.5 ± 18.6	0.59
EPICES	17.8 ± 17.9	8.4 ± 11.0	0.21
DFMT index	20.9 ± 8.4	17.1 ± 6.8	0.20
Gingivitis,* n*	9	7	0.72
Periodontitis , *n*	10	8	0.71
Thick gingival biotype,* n*	4	5	0.70
Add-GOHAI	51.5 ± 6.8	50.2 ± 9.3	0.69
SC-GOHAI	3.3 ± 3.0	3.4 ± 3.4	0.97

Data are given as absolute numbers or mean ± s.d. For statistical tests see ‘Methods’.

UNL: fold of the upper normal limit.

## Discussion

We conducted the largest study to date accurately assessing at the same time clinical and radiological parameters of the dental and periodontal state as well as OHRQoL in acromegalic patients, revealing new concepts in this field.

Concerning dental state, DMFT was previously determined in the French population for the 35- to 44-year age group (20) and for the 65- to 74-year age group (21) as it is highly dependent of age. Seven acromegalic patients between 35 and 49 years old were included in our study and their DMFT index were 14.6 ± 7.1 vs 14.6 ± 6.0 in the French population (20) (we assumed that dental state in 44- to 49-year-old patient is close from the one of 35- to 44-year-old patients). Eight acromegalic patients between 65 and 74 years old were included in our study and their DMFT index were 21.3 ± 4.5 vs 23.3 ± 7.4 in the French population (21). Despite the global similarity of these DMFT indexes, when compared to data reported in the French population (20, 21), we could note that: (1) tooth decay appeared less frequent in acromegalic patients in our study and seemed not impacted by age (young: 0.4 ± 0.5 vs 1.2 ± 2.0; old: 0.4 ± 0.5 vs 1.1 ± 2.0), (2) in the older age group (35–44 years), there were more filled teeth (12.6 ± 3.3 vs 5.2 ± 5.1) and less missing teeth (8.3 ± 4.2 vs 16.9 ± 10.5) in acromegalic patients of our study. These observations suggest a similar incidence of dental pathology in acromegalic patients than in French population with more dental cares carried out in acromegalic patients, concordantly with the low EPICES score found in our population. However, they should be interpreted with care as data for the French population were collected more than 20 years before our study. Because of those limits, our finding of a normal dental condition in acromegalic patients has to be confirmed by a specifically designed prospective controlled study.

Prevalence of periodontitis was estimated at 29% in the French population (22) vs 18/29 patients in our study, but very diverse results could be found between different studies made in European countries (range 13–82%) illustrating that this parameter is difficult to assess and strongly influenced by age, socio-economic situation and health care organization (23). In our study, gingivitis and gingival bleeding frequency looked similar to that observed in daily practice. Most cases of periodontitis were localized chronic adult periodontitis likely due to a local factor leading to difficulty of teeth cleaning (inadequate artificial crown or tooth filling). Moreover, there was no case of severe periodontitis. Three previous controlled studies found a decreased frequency or severity of periodontitis in acromegalic patients compared to controls (9, 10, 11). One suggested explanation was a positive role of GH and IGF-1 on inflammation rather than on bone metabolism based on the dosage of gingival crevicular fluid biomarkers (11). However, we could wonder if GH and IGF-1 effects on soft tissues could participate in periodontal disease protection in acromegaly as 9/29 of our patients had a thick gingival biotype. Though there is no data on the prevalence of thick gingival biotype in the French population, it seems that gingival biotype was globally thicker in acromegalic patients than what is observed in daily practice. Thick gingival biotype is associated with periodontal health and a better outcome for periodontal treatment (24).

But trophic action of GH and IGF-1 on periodontal tissues seemed complex. Indeed, we found no case of diffuse gingival hypertrophy (only one case of bilateral palatal tuberous outgrowth). One study described mild to moderate gingival ‘enlargement’ in 8 acromegalic patients out of 11 examined (8) but it was not reported if this term means thick gingival biotype or gingival hypertrophy and if periodontitis was associated. In the same way, although there is preclinical evidence of a trophic role of acromegaly on cementum (25) and two published cases reports mentioning radiological hypercementosis on molars of acromegalic patients (12, 13), systematic orthopantomogram revealed only two cases of hypercementosis, each affecting only one tooth per patient. Hypercementosis could result from local factors such as a chronic periapical infection, lack of function due to unopposed teeth and trauma from abnormal dental occlusion (26). As occlusion disorder was present in a majority of acromegalic patients, it is likely that local conditions rather than hormonal effects contribute to hypercementosis development in acromegaly. Further, a direct role of IGF-1 excess would affect all the teeth equally, leading to diffuse hypercementosis visible on X-ray examinations.

Concerning bone modifications, apart from the well-described jaw enlargement leading to occlusion disorders (5), we report for the first time the presence of bulky OBO in 11/29 acromegalic patients while this is quite rare in the general population. The prevalence of obvious OBO has never been investigated in the French population, but the observation of such a high frequency in a series of patients is very unusual in daily practice. Reported prevalence of OBO is very variable in the literature, from 1 to 60% for each form of tori (mandibular and maxillary) (27) and from 2.0 to 26% for vestibular exostosis (28, 29). This discrepancy is related to a probable impact of ethnicity but also to the means used to assess OBO presence (inspection, palpation or plaster casts examination). Numerous studies take into account very small OBO which are much more frequent than the bulky obvious OBO we are reporting here. For example in a recent Lithuanian study using plaster casts, maxillary tori were present in 1.8% of subject and mandibular tori in 57%, but more than 50% of these lesions measured less than 2 mm and only 6% were over 4 mm (30). In our study, we noticed only lesions over 3 mm and half acromegalic patients with OBO had lesions over 6 mm. In a Moroccan study using only oral examination in 353 patients, mandibular tori over 3 and over 6 mm were found in 1.1 and 0.3% of patients, respectively, maxillary tori over 3 and over 6 mm were found in 0.3 and 0% of patients respectively, and vestibular exostosis in 2.0% of patients, but never located on mandible (29) while this was observed for 2/29 acromegalic patients. Up to now, no hormonal cause was identified for OBO development but a favoring role of occlusion disorder was suggested (29). There was no link between the presence of OBO and acromegaly parameters, but this could be due to an insufficient number of cases. Our results cannot discern if GH and IGF-1 excess causes appearance of OBO or if it induces the enlargement of small preexisting exostosis but some cases were noted after a short evolution of the disease. As a consequence, presence of obvious bulky OBO in a patient visiting a dentist for occlusive disorder should prompt investigations to rule out acromegaly.

To our knowledge, OHRQoL has never been investigated in acromegaly before. In our study, the mean Add-GOHAI and SC-GOHAI scores was 50.9 ± 8.0 and 3.4 ± 3.1, respectively, while they were 46.4 ± 9.5 and 4.5 ± 3.2, respectively, in the French population reference study (19). But if OHRQoL scores seem slightly better in acromegalic patients, some confounding factors in the reference study can explain this difference (socio-economically disadvantaged patients, looking for dental care). Other studies in Western Europe focalized on elderly population and reported a mean Add-GOHAI score of 52.8 in Germany (31), 49.8 in Sweden (32) and 51.5 in the Netherlands (33). A recent internet-based study in France found a mean Add-GOHAI of 54.5 ± 4.3 in a large representative population, but also demonstrated a positive correlation between Add-GOHAI and age (34). In this study, the proportion of Add-GOHAI scores ≥ 57 (satisfactory), between 50 and 57 (moderate) and ≤ 50 (poor OHRQoL) was 40, 42 and 18%, respectively, while they were 8/26, 9/26 and 9/26 in acromegalic patients, respectively, indicating that acromegaly has a negative impact on OHRQoL in a subset of patients. The qualitative analysis of the GOHAI questionnaire reveals that acromegalic patients often experience pain and discomfort and are frequently concerned about their oral health. This could be due to symptoms linked to occlusion disorders. However, we could not find any association between OHRQoL alteration and patient characteristics, acromegaly parameters or objective oral manifestations. On the contrary, acromegalic patients with poor OHRQoL had a worse acromegaly-specific HRQoL, according to AcroQoL. It has already been shown that OHRQoL (assessed by SC-GOHAI) is lower in elderly people with alteration of well-being and depression (31), two factors also assessed by AcroQoL. A recently published postal survey in a large cohort of acromegalic patients showed that patients reporting oro-dental pathologies had a worse global quality of life (assessed by SF-36 scale) (35), which highlights the importance of oral health for patients well-being. The absence of link between disease control and OHRQoL could be explained by irreversible modifications, such as occlusive disorder, in the same way, that impaired disease-specific quality of life persists after long term control of acromegaly (36).

## Conclusion

To our knowledge, this is the first prospective study to assess at the same time dental and periodontal state as well as OHRQoL in the course of acromegaly. This enabled us to highlight that acromegalic patients present a globally satisfactory dental and periodontal condition, quite similar to that of the general population of the same country. Acromegaly does not appear to be a cause of generalized gingival hypertrophy and hypercementosis but could protect from a severe periodontal disease by conferring more robust periodontal tissues and thicker gingival biotype. Yet, we showed that more than 1/3 acromegalic patients have a poor satisfaction of OHRQoL which did not seem to be linked with disease control nor with objective oral impairment, but rather with acromegaly related alteration of quality of life. Finally, we discovered that bulky OBO are present in 11/29 of patients but we could not find any association between their appearance and patients’ characteristics. However, this obvious sign may appear early in the disease history and could contribute to reduce the diagnostic latency in acromegaly. Taking our results into account, we advocate an annual oral examination for acromegalic patients. More specifically designed prospective controlled studies are, however, necessary to precise our findings.

## Declaration of interest

The authors declare that there is no conflict of interest that could be perceived as prejudicing the impartiality of the research reported.

## Funding

The authors acknowledge the financial support received as a donation (Number: DG CPL F 0002 V1) from Novartis Pharma SAS – rare diseases.
